# Westminster CAMHS Happy Doc Spread Reducing Initial Assessment Time: Freeing Up Time for Treatment

**DOI:** 10.1192/bjo.2025.10365

**Published:** 2025-06-20

**Authors:** Harry Fitzpatrick, Sophie Parry-Williams, Annie Waterfield, Marissa Lee, Tami Kramer

**Affiliations:** 1 CNWL, London, United Kingdom; 2 West London NHS Trust, London, United Kingdom

## Abstract

Aims:

Main outcome: To reduce the total time taken for Initial Assessments (IAs) in CAMHS by 10% by January 2025.

Process measure: To reduce time taken to complete the Initial Assessment Form and Care Plan letter.

Balance measures: To improve service user experience of the assessment process; to improve clinician experience of completing Initial Assessments (IAs).

**Methods:** PDSA 1: Developmental and medical history form collected ahead of Initial Assessment.

PDSA 2: Happy Doc Initial Assessment Proforma and automated Care Plan Letter.

PDSA 3: Dictation software.

PDSA 4: Locally developed wild card PDSA – parents offered a “Pre-assessment” session, initially trialled as a phone session (PDSA 4 i) and subsequently in person (PDSA 4 ii).

A parent QI Team member offered Expert by Experience advice on design and implementation. Parent views on the Care Plan Letter and Pre-assessment session are being collected by questionnaire. Qualitative and quantitative data has been collected from clinicians on each PDSA cycle.

**Results:** PDSA 1: Medical & Developmental History forms were not returned to the clinic ahead of assessment. To implement differently within PDSA 4.

PDSA 2: Process measure indicated 35% reduction in Time to complete IA Form and Care Plan Letter.

PDSA 3 and 4: No change to Total Initial Assessment Time yet. Possible early suggestion of reduced variation between assessments.

Parent feedback: Face to face Pre-assessment Clinic rated as positive and useful experience. Parents appreciate a space to share information without their children present.

Clinician feedback: “The assessment has felt so much quicker with the Pre-assessment session. Usually it feels unfinished after 2 appointments but I feel that I have enough information to conclude the assessment.”

**Conclusion:** The significant reduction of time from PDSA 2 (35%) reflected by the Process Measure has not yet impacted significantly on the Total IA Time.

Subsequent introduction of dictation software (DragonMedical1) was difficult for clinicians, with low satisfaction and negative impact on time. With further use and individual adaptation, feedback improved. Implementation of a technology to aid workflow may require more time for learning before benefits become evident.

Parent engagement in telephone Pre-assessment Clinic (PDSA 4i) was poor. Following further iteration to in-person format (PDSA 4ii), engagement and feedback have improved.

The Pre-assessment Clinic has reduced assessment related tasks for clinicians who report this as a positive experience.

Positive staff stories about the Happy Doc Initial Assessment Proforma and Care Plan Letter led to the whole service deciding to adopt it.

